# Electroacupuncture of Baihui and Shenting ameliorates cognitive deficits *via* Pten/Akt pathway in a rat cerebral ischemia injury model

**DOI:** 10.3389/fneur.2022.855362

**Published:** 2022-08-19

**Authors:** Kaiqi Su, Wenxue Hao, Zhuan Lv, Mingli Wu, Jieying Li, Yanchao Hu, Zhenhua Zhang, Jing Gao, Xiaodong Feng

**Affiliations:** ^1^Department of Rehabilitation Medicine, Henan University of Chinese Medicine, Zhengzhou, China; ^2^Rehabilitation Center, The First Affiliated Hospital of Henan University of Chinese Medicine, Zhengzhou, China; ^3^Department of Rehabilitation, The Third Affiliated Hospital of Zhengzhou University, Zhengzhou, China; ^4^College of Basic Medicine, Zhejiang Chinese Medical University, Hangzhou, China

**Keywords:** cerebral ischemia stroke, proteomics, Baihui, Shenting, acupuncture

## Abstract

Cerebral ischemic stroke is a huge threat to the health and life of many people. Electroacupuncture (EA) at Baihui (GV20) and Shenting (GV24) acupoints can notably alleviate cerebral ischemia/reperfusion injury (CIRI). However, the molecular basis underlying the effectiveness of EA at the GV20 and GV24 acupoints for CIRI remains largely unknown. Our present study demonstrated that EA treatment at the GV20 and GV24 acupoints markedly alleviated middle cerebral artery occlusion/reperfusion (MCAO/R)-induced cognitive deficits and cerebral infarction in rats. Proteomics analysis revealed that 195 and 218 proteins were dysregulated in rat hippocampal tissues in the MCAO/R vs. sham group and thhhe EA vs. MCAO/R group, respectively. Moreover, 62 proteins with converse alteration trends in MCAO/R vs. sham and EA vs. MCAO/R groups were identified. These proteins might be implicated in the EA-mediated protective effect against MCAO/R-induced cerebral injury. GO enrichment analysis showed that 39 dysregulated proteins in the MCAO/R vs. sham group and 40 dysregulated proteins in the EA vs. MCAO/R group were related to brain and nerve development. Protein–protein interaction analysis of the abovementioned dysregulated proteins associated with brain and nerve development suggested that Pten/Akt pathway-related proteins might play major roles in regulating EA-mediated protective effects against MCAO/R-induced brain and nerve injury. Western blot assays demonstrated that Pak4, Akt3, and Efnb2 were expressed at low levels in the MCAO/R group vs. the sham group but at high levels in the EA group vs. the MCAO/R group. In conclusion, multiple proteins related to the protective effect of EA at the GV20 and GV24 acupoints against CIRI were identified in our study.

## Introduction

Stroke is the second leading cause of death worldwide, accounting for more than 11% of total deaths. It was estimated that ~12 million new stroke cases and 6.55 million deaths due to stroke occurred globally in 2019 (1). The incidence and mortality rates of stroke are higher in low-income and middle-income countries than in high-income countries (1, 2). It has been reported that many metabolic, behavioral, and environmental factors (e.g., hypertension, high body-mass index, physical inactivity, and air pollution) can increase the risk of stroke (2–4). Stroke can be classified into two major types, namely, hemorrhagic stroke and ischemic stroke, and ischemic stroke is responsible for the majority of stroke cases (5, 6). Over the past decades, the middle cerebral artery occlusion/reperfusion (MCAO/R) animal models have been widely used to explore the pathogenesis of ischemic stroke and address the molecular mechanisms of drugs and agents in the treatment of ischemic stroke due to advantages such as mimicking human ischemic stroke, high repeatability, high controllability on reperfusion, and no craniectomy (7, 8).

Acupuncture has been widely used for the treatment of multiple diseases, namely, central nervous system diseases in some countries, especially in China (9–11). Acupuncture and electroacupuncture (EA) can notably alleviate poststroke cognitive and locomotor function impairments (12, 13). However, acupuncture is infrequently accompanied by some adverse events such as subarachnoid hemorrhage, pneumothorax, and faintness (14, 15). Thus, a growing number of scientists paid attention to assessing acupuncture effectiveness and exploring the underlying biological basis of acupuncture in recent years.

Baihui (GV20) and Shenting (GV24), two frequently used acupoints in patients with cognitive impairment, can markedly improve the cognitive functions of these patients (16–18). Some prior studies demonstrated that EA stimulation at GV20 and GV24 acupoints could notably ameliorate MCAO/R-induced cognitive deficits and behavioral dysfunctions (19–21). For instance, Liu et al. showed that MCAO/R surgery could induce rat neurological deficits, increase rat escape latency time, and reduce the frequency of rat crossing over the platform location partially by promoting cerebral cortex cell apoptosis, while these effects were noticeably alleviated after EA treatment at the GV20 and GV24 acupoints (19). EA stimulation at the GV20 and GV24 acupoints led to a notable reduction in rat neurological deficit scores, cerebral infarct volume, and escape latency time and a marked increase in hippocampal synaptic plasticity and the number of times the rats passed through the platform area by facilitating the expression of the brain-derived neurotrophic factor (22). However, these studies mainly focused on the roles and certain molecular mechanisms of EA at the GV20 and GV24 acupoints in the MCAO/R model. To gain more comprehensive knowledge about the scientific and molecular bases by which EA at the GV20 and GV24 acupoints could frequently ameliorate MCAO/R-induced brain dysfunctions, tandem mass tag (TMT)-based proteomics analysis was performed in the hippocampal tissues of rats in the sham, MCAO/R model, and EA treatment groups to identify proteomics alterations induced by MCAO/R or EA stimulation at the GV20 and GV24 acupoints. Moreover, some proteins related to brain and nerve development that might play major roles in MCAO/R-induced cerebral injury and EA-mediated protective effects against MCAO/R-induced cerebral injury were screened out.

## Materials and methods

### Animals

Healthy adult male-specific-pathogen-free (SPF) Sprague–Dawley rats (260 ± 20 g) were purchased from Huaxing Experimental Animal Center (License No.: SCXK (YU) 2019–0002, Zhengzhou, China). Rats were raised in the Central Laboratory of the First Affiliated Hospital of the Henan University of Chinese Medicine with free access to water and food. Rats were allowed to acclimatize to the new surroundings for 1 week. Our animal experiments were approved by the Animal Ethics Committee of the First Affiliated Hospital of the Henan University of Chinese Medicine (Approval No. YFYDW2019037).

### Morris water maze (MWM) assay

To exclude rats with congenital dementia from our project, the spatial learning and memory abilities of rats after 1 week of acclimatization were assessed by MWM assay as previously described (23, 24). A circular water tank (diameter: 1.6 m; height: 0.5 m) was used in the MWM assay. The tank was filled with water (water depth: 30 cm; water temperature: 21–25°C) before training. The water tank was divided into four equal quadrants. A colorless transparent circular escape platform (diameter: 12 cm; height: 29 cm) was placed in the center of one quadrant and submerged ~1 cm below the water surface. Visual references were hung at different sites around the maze. Before animal experiments, rats with congenital dementia were screened out by place navigation tests after three rounds of training. For each round of training, rats were initially placed into the water in quadrant 1 and then sequentially placed into the other three quadrants in a clockwise direction. Rats were given 90 s to find the hidden platform. If rats could find the platform within 90 s and stay on the platform for over 3 s, the time that they spent finding the escape platform (i.e., escape latency time) was recorded. If rats could not find the platform within 90 s, they were guided to the escape platform and allowed to rest for 10–30 s before the subsequent trials, and the escape latency time was recorded to be 90 s. Rats that did not find the platform within 90 s were considered abnormal in intelligence and were excluded from our project. A total of 72 rats were used in the subsequent experiments. Among these 72 rats, 12 rats were assigned to the control group and 15 rats were allocated to the sham group. The other 45 rats were used for the construction of the MCAO/R model. These rats were randomly assigned.

Additionally, the effect of EA treatment on the spatial learning and memory abilities of MCAO/R rats was assessed by a spatial navigation test on days 9–13 after EA treatment. Rats were subjected to two rounds of training each day. On day 14 after EA treatment, the spatial memory abilities of rats were further examined by a spatial probe test. Briefly, the platform was removed after spatial navigation experiments. Next, the rats were placed in the water and the number of times the rats crossed the original platform position within 90 s was recorded.

### MCAO/R rat model construction and neurological deficit assessment

After 12 h of fasting, rats in the sham (*n* = 15) and MCAO/R (*n* = 45) groups were anesthetized using anesthetic pentobarbital sodium (50 mg/kg body weight, Sigma-Aldrich, St. Louis, MO, USA) by intraperitoneal injection. Then, MCAO/R rat models were constructed as previously reported (25–27). Briefly, rats (*n* = 45) were fixed onto operating tables in the supine position after anaesthetization. Then, the left common carotid artery (CCA), the external carotid artery (ECA), and the internal carotid artery (ICA) were exposed through a neck longitudinal incision. The proximal parts of the ECA and the CCA were ligatured, and a slip knot was reserved at the bifurcation site of the CCA near the ICA and the ECA. The distal end of the ICA was clamped using the artery clip. The MCAO monofilament (type: 2636A4, Beijing Xinong Technology Co., LTD, China) was inserted into the ICA (insertion depth: 18–20 mm) through a CCA “V”-shaped incision ~1 cm away from the bifurcation of the ECA and the ICA until mild resistance was felt. Approximately, 1 cm of MCAO monofilament was reversed outside. The CCA and the MCAO monofilaments were ligated at the reversed slipknot to fix the MCAO monofilament. Next, the neck incision was sutured and the end of the MCAO monofilament was labeled using a black marker pen. After 2 h of ischemia, the MCAO monofilament was withdrawn to achieve reperfusion. Rats in the sham group (n = 15) only suffered from exposure to CCA, ECA, and ICA. After surgery, all rats were intraperitoneally injected with gentamicin (20,000 units/rat) for three consecutive days and intramuscularly injected with furosemide (0.1 mg/kg). The electric incandescent lamp was used to control the body temperature of rats during the MCAO procedure. After anesthetization and surgery, rats were placed in a large box containing an electric heating blanket to control their body temperature. Nine rats died after MCAO/R surgery. Of these nine rats, five died during monofilament insertion, and four died after the removal of the monofilaments. The survival rate of the MCAO/R rats was 80.0% (36/45).

At 2 h after surgery, the neurological function impairment patterns of rats were evaluated by the Zea-Longa score system as previously described (25). The neurological deficit scores were examined by a researcher who was blinded to our experimental designs. The details of the Zea-Longa score system were as follows: a score of 0 did not show neurological injury symptoms; a score of 1 signified a mild focal neurological deficit with the symptom of a failure to extend the surgery contralateral forepaw fully; a score of 2 represented a moderate focal neurological deficit with the symptom of circling to the contralateral side; a score of 3 indicated a severe focal neurological deficit with the symptom of falling to the contralateral side; and a score of 4 denoted a serious neurological deficit characterized by the inability to walk spontaneously and reduction/loss of consciousness. The MCAO/R model was regarded to be successfully established in rats with scores of 1–3. Rats with scores of 0 (*n* = 4) and 4 (*n* = 2) were excluded. Finally, 30 rats in the MCAO/R model construction group were enrolled in our subsequent experiments and randomly divided into the MCAO/R group (*n* = 15) and the electroacupuncture (EA) group (*n* = 15).

### EA treatment

**Figure 1:**
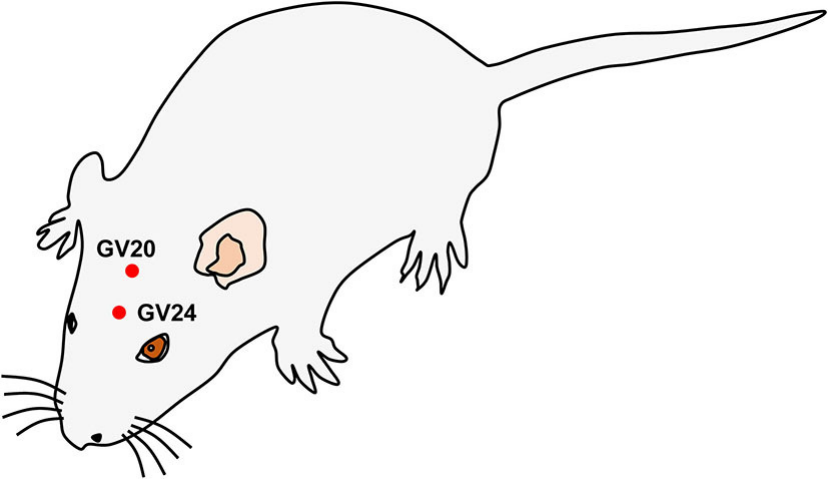
Schematic diagram of Baihui (GV20) and Shenting (GV24) acupoints.

Day 2 after surgery, rats in the EA group were subjected to EA treatment at the GV20 and GV24 acupoints one time a day for a total of 14 days using the Huatuo electroacupuncture treatment device (Suzhou Medical Appliance Factory, Suzhou, China). The needles were inserted into the GV20 and GV24 acupoints at an angle of 45° and a depth of 2 mm. GV20 is in the middle of the parietal bone, while GV24 is on the anterior midline, in front of the frontal–parietal bone junction (Figure 1). The parameters of the Huatuo electroacupuncture treatment device were as follows: dilatational wave, 1–20 Hz of frequency, 1–3 mA of electric current intensity, and 30 min of duration time per day. On Day 7 or 14 after EA treatment, the neurological deficit patterns of rats were also assessed by the Zea-Longa score system.

### Tissue sample preparation

Three rats per group were randomly selected from the control, sham, MCAO/R, or EA groups. Then, the whole brains of these rats were isolated for a subsequent triphenyl tetrazolium chloride (TTC) staining assay as previously described (28). Briefly, rat hearts were exposed after anesthesia and then perfused with 0.9% normal saline. Rats were sacrificed by cervical dislocation when the colorless liquid flowed from the right auricle incision and then the brains were quickly isolated. The other rats were decapitated after anesthesia. Next, the brains and hippocampi of these rats were quickly isolated on ice, snap-frozen in liquid nitrogen, and stored at −80°C.

### TTC staining analysis

After isolation, rat brains were frozen for ~20 min at −20°C and sectioned into 2 mm coronal slices. Next, the sections were stained with 2% TTC solution for 30 min at 37°C in the dark and then imaged. The areas of cerebral infarction were manifested by white staining, while the areas with no cerebral infarction were stained red. The volume of cerebral infarction was analyzed by Image-Pro Plus software (Media Cybernetics, Silver Spring, MD, USA).

### TMT-based proteomics analysis

Whole hippocampal tissues from the Sham (*n* = 9), MCAO/R (*n* = 9), and EA (*n* = 9) groups were used in the TMT-based proteomics analysis. Each group contained nine samples, and three samples per group were mixed into a proteomics specimen. In the proteomics analysis, the sham group samples were named C1 (TMT label no. 126), C2 (TMT label no. 127N), and C3 (TMT label no. 127C). The MCAO/R model group samples were named M1 (TMT label no. 128N), M2 (TMT label no. 128C), and M3 (TMT label no. 129N). The EA treatment group samples were termed T1 (TMT label no. 129C), T2 (TMT label no. 130N), and T3 (TMT label no. 130C). Proteins were regarded as significantly differentially expressed at an upregulated ratio of ≥ 1.2 or a downregulated ratio of ≤ 0.83 and an adjusted *P*-value of ≤ 0.05. The detailed experimental procedures of TMT-based proteomics analysis are shown in Supplementary file 1.

### Bioinformatics analysis

Venn diagram analysis was performed using the Jvenn website (http://jvenn.toulouse.inra.fr/app/example.html) (29). Protein–protein interaction (PPI) networks were established using STRING (version: 11.5) (https://cn.string-db.org/) (30, 31). K-means clustering analysis was performed using the MeV (MultiExperiment Viewer) program (https://mev.tm4.org/) (32).

### Western blot assay

Protein was extracted from hippocampal tissues of the sham (*n* = 3), MCAO/R (*n* = 3), and EA (*n* = 3) groups using RIPA buffer (Beijing Solarbio Science & Technology Co., Ltd., Beijing, China) supplemented with the protease inhibitor phenylmethanesulfonyl fluoride (PMSF). The protein concentration was determined using the BCA Protein Assay Kit (Solarbio Science & Technology Co., Ltd., Beijing, China). Next, an equal amount of protein (40 μg) was separated by 10% SDS-PAGE and transferred onto 0.22 μm polyvinylidene fluoride (Millipore, Bedford, MA, USA). After the blockade of nonspecific protein signals using 5% skimmed milk, the membranes were hybridized overnight with primary antibodies against Pak4 (1:1,000; ImmunoWay Biotechnology Company, Plano, TX, USA), Akt3 (1:1,000; ImmunoWay Biotechnology Company, Plano, TX, USA), Efnb2 (1:1,000; ImmunoWay Biotechnology Company, Plano, TX, USA), and Gapdh (1:10,000; ImmunoWay Biotechnology Company, Plano, TX, USA) at 4°C. On the next day, the membranes were probed for 1 h with goat–anti-rabbit or goat–anti-mouse (1:10,000; ImmunoWay Biotechnology Company, Plano, TX, USA) secondary antibodies conjugated with horseradish peroxidase at room temperature. Finally, protein bands were detected using the ECL Western Blotting Substrate (Beijing Solarbio Science & Technology Co., Beijing, China).

### Statistical analysis

Statistical analysis was performed using GraphPad Prism software (version 7, La Jolla, CA, USA), and the results are shown as the mean ± standard deviation. The differences among groups were analyzed using one-way or two-way analysis of variance (ANOVA) and Tukey's test. The differences were defined as statistically significant when the *p-value* was < 0.05.

## Results

### EA treatment at the GV20 and GV24 acupoints notably ameliorated neurological deficits in MCAO/R rats

In this study, the neurological functions of rats in the control, sham, MCAO/R, and EA groups were assessed by the Zea-Longa score system. The results showed that there were no neurological deficit symptoms in the control and sham rats, while certain neurological deficits were observed in rats in the MCAO/R and EA groups at 2 h after surgery (Table 1). There was no significant difference (*P* > 0.05) in the neurological deficit scores between the MCAO/R and EA groups at 2 h after modeling. However, the number of rats with lower neurological deficit scores (scores 0 and 1) was notably increased in the EA group at 7 or 14 days after EA treatment compared to the MCAO/R model group (Table 1), suggesting that EA treatment could markedly alleviate MCAO/R-induced rat neurological injury.

**Table 1:** Neurological deficit scores of rats in the four groups.

**Groups**	**Total number**	**Detection time**	**Score 0**	**Score 1**	**Score 2**	**Score 3**	**Score 4**
Control	12	2 h after surgery	12	0	0	0	0
		7 days after EA treatment	12	0	0	0	0
		14 days after EA treatment	12	0	0	0	0
Sham	15	2 h after surgery	15	0	0	0	0
		7 days after EA treatment	15	0	0	0	0
		14 days after EA treatment	15	0	0	0	0
MCAO/R	15	2 h after surgery	0	0	5	10	0
		7 days after EA treatment	0	2	7	6	0
		14 days after EA treatment	0	5	8	2	0
EA	15	2 h after surgery	0	0	7	8	0
		7 days after EA treatment	1	8	6	0	0
		14 days after EA treatment	6	8	1	0	0

### EA treatment at the GV20 and GV24 acupoints markedly improved the spatial learning and memory abilities of MCAO/R rats

**Figure 2:**
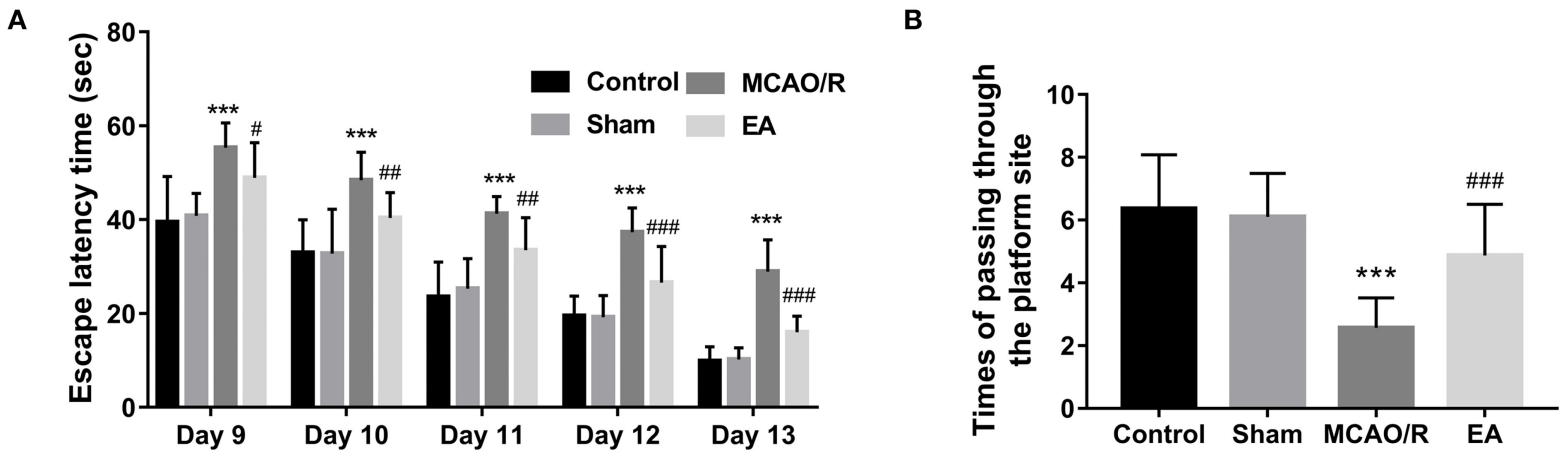
EA treatment at the GV20 and GV24 acupoints markedly improved the spatial learning and memory abilities of MCAO/R rats. **(A)** The time that the rats spent finding the escape platform was examined by the MWM assay at 9–13 days after EA treatment. **(B)** The escape platform was removed on day 14 after EA treatment. Next, the number of times the rats passed through the platform position within 90 s was recorded. There were 12, 15, 15, and 15 rats in the control, sham, MCAO/R, and EA group, respectively. Compared with the sham group, ***P < 0.001; compared with the MCAO/R group, #*P* < 0.05, ##*P* < 0.01, ###*P* < 0.001.

The MWM assay was performed to examine the effect of EA treatment on the spatial learning and memory behaviors of MCAO/R rats. As seen in Figure 2A, MCAO/R model rats spent more time escaping latency than rats in the sham group. Moreover, the spatial probe assay showed that there was a marked decrease in the frequency of swimming across the platform within 90 s in the MCAO rats vs. the sham group (Figure 2B). These data showed that MCAO/R rats had impaired spatial learning and memory behaviors compared to the sham group. Additionally, EA treatment noticeably reduced the escape latency time of MCAO/R rats from Day 9 to Day 13 after stimulation (Figure 2A). After EA treatment, MCAO/R rats crossed the platform positions more times than untreated MCAO/R rats (Figure 2B). These outcomes suggested that EA treatment at the GV20 and GV24 acupoints notably ameliorated the spatial learning and memory impairments of MCAO/R rats.

### EA treatment at the GV20 and GV24 acupoints alleviated MCAO/R-induced rat cerebral infarction

**Figure 3:**
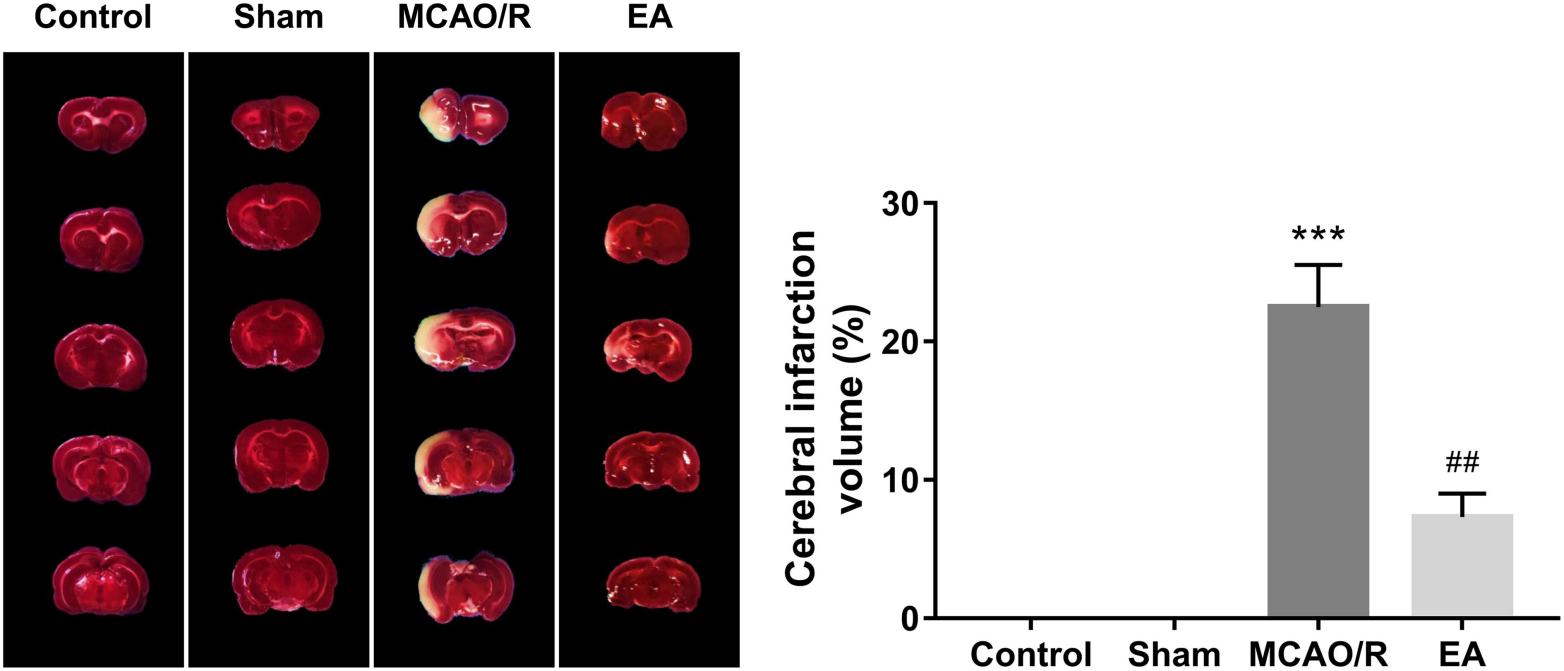
EA treatment at the GV20 and GV24 alleviated MCAO/R-induced rat cerebral infarction. On Day 14 after EA treatment, the whole brains of rats were isolated and then stained with TTC. The cerebral infarction areas were stained white. Each group contained three brain samples from three rats, and five coronal sections were obtained from each brain. Compared with the sham group, ***P < 0.001; compared with the MCAO/R group, #*P* < 0.05, ##*P* < 0.01, ###*P* < 0.001.

TTC staining analysis further revealed that MCAO/R rats developed obvious cerebral infarction, while the volume of cerebral infarction was notably reduced in MCAO/R rats after EA treatment compared with untreated MCAO/R rats (Figure 3), suggesting that EA stimulation could markedly relieve cerebral infarction in MCAO/R rats.

### Identification of differentially expressed proteins in the MCAO/R vs. Sham and EA vs. MCAO/R groups

**Figure 4:**
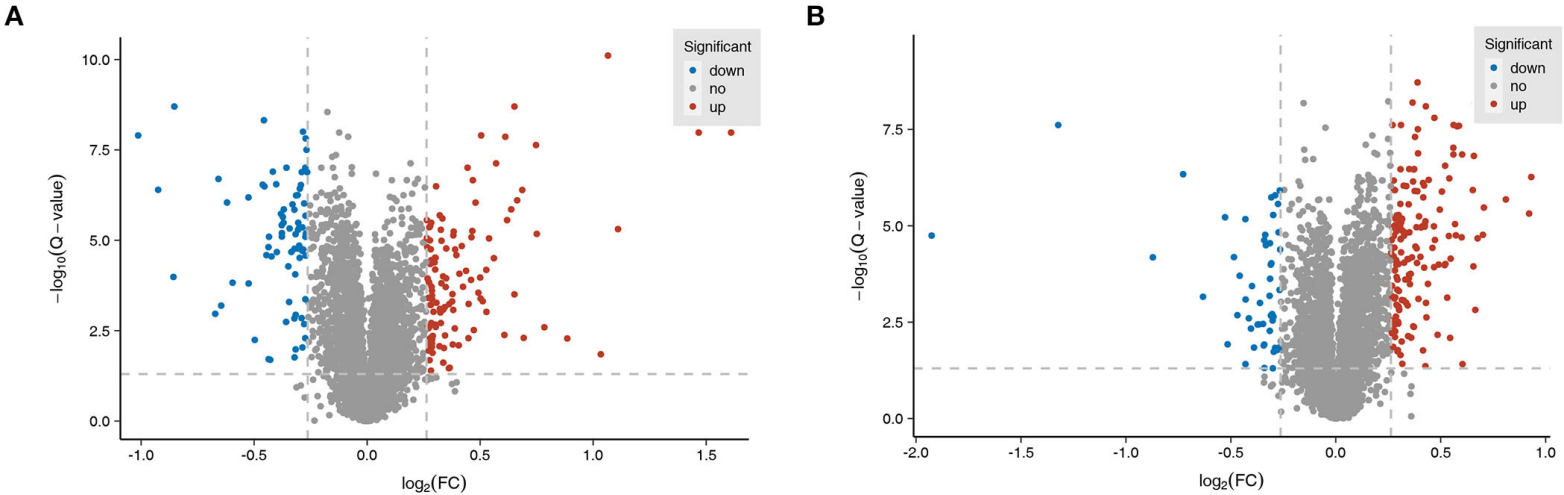
Identification of dysregulated proteins and biological processes related to MCAO/R-induced brain injury in rats. **(A)** The volcano plot of differentially expressed proteins in the MCAO/R vs. sham group. **(B)** The volcano plot of differentially expressed proteins in the EA vs. MCAO/R group. Each group contained nine hippocampal tissue samples isolated from nine rats, and three samples per group were mixed into a proteomics specimen.

TMT-based proteomics analysis showed that the expression levels of 75 proteins were notably downregulated and the expression levels of 120 proteins were markedly upregulated in the MCAO/R group vs. the sham group (Supplementary Table 1). The volcano plot of differentially expressed proteins in the MCAO/R vs. sham group is shown in Figure 4A. Moreover, a total of 218 differentially expressed proteins (50 downregulated, 168 upregulated) were identified in the EA treatment group compared with the MCAO/R group (Supplementary Table 2). The volcano plot of differentially expressed proteins in the EA vs. MCAO/R group is shown in Figure 4B.

### Identification of proteins with adverse alteration trends in the MCAO/R vs. sham and EA vs. MCAO/R groups

**Figure 5:**
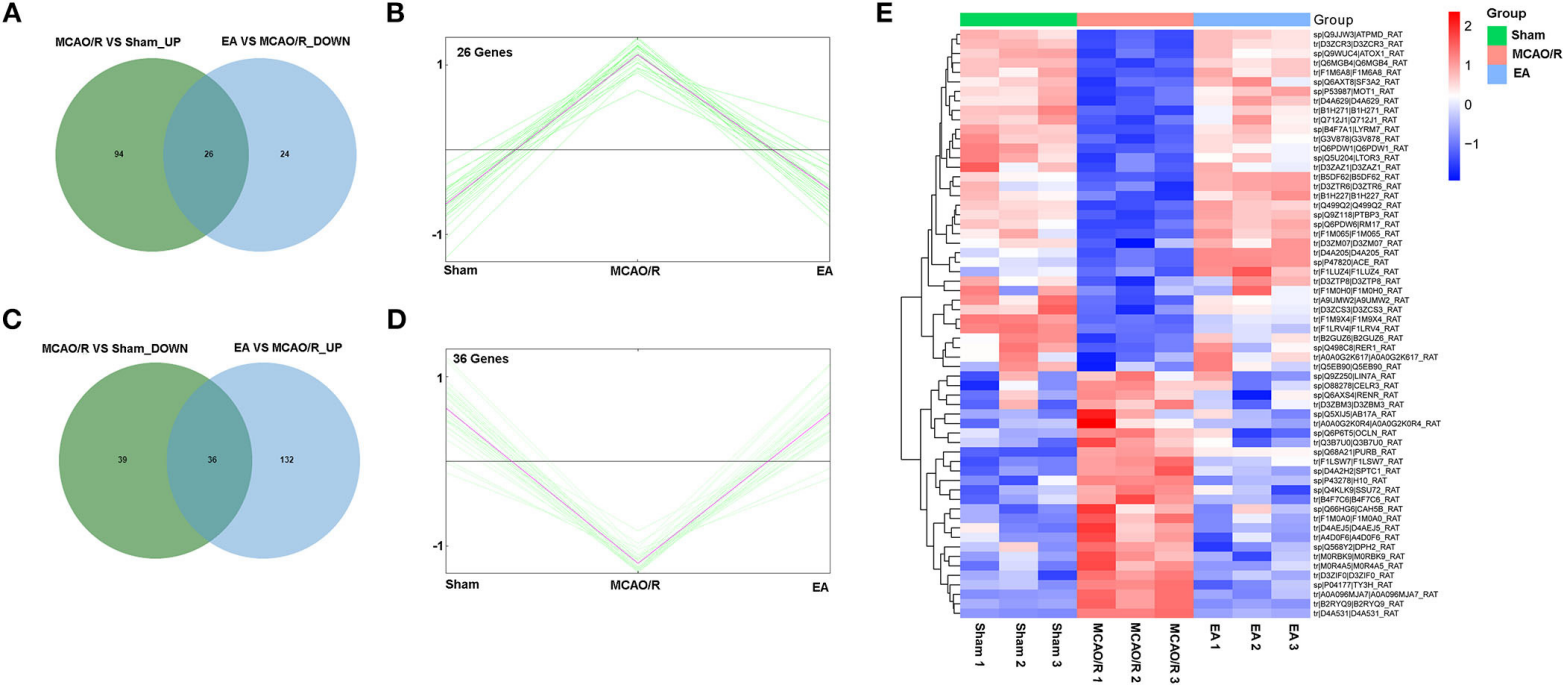
Identification of proteins with adverse alteration trends in the MCAO/R vs. sham and EA vs. MCAO/R groups. **(A)** Jvenn analysis of upregulated proteins in the MCAO/R vs. sham group and downregulated proteins in the EA vs. MCAO/R group. **(B)** K-means clustering plot of the expression profiles of proteins that were upregulated in the MCAO/R group vs. the sham group and downregulated in the EA group vs. the MCAO/R group. **(C)** Jvenn analysis of downregulated proteins in the MCAO/R vs. sham group and upregulated proteins in the EA vs. MCAO/R group. **(D)** K-means clustering plot of the expression profiles of proteins that were downregulated in the MCAO/R vs. sham group and upregulated in the EA vs. MCAO/R group. **(E)** Heatmap of 62 proteins with converse alteration trends in the MCAO/R vs. sham group and the EA vs. MCAO/R group. Each group contained nine hippocampal tissue samples isolated from nine rats and three samples per group were mixed into a proteomics specimen.

Next, 26 proteins that were notably upregulated in the MCAO/R vs. sham group and markedly downregulated in the EA vs. MCAO/R group were screened out by jvenn analysis and are shown in Figure 5A and Supplementary Table 3. The K-means clustering plots of the expression profiles of these 26 proteins are shown in Figure 5B. Additionally, 36 proteins that were noticeably downregulated in the MCAO/R vs. sham group and upregulated in the EA vs. MCAO/R group were filtered out by jvenn analysis and are shown in Figure 5C and Supplementary Table 3. The K-means clustering plots of these 36 proteins are shown in Figure 5D. The heatmap of these 62 proteins with converse alteration trends in the MCAO/R vs. sham and EA vs. MCAO/R groups is shown in Figure 5E. As seen in Figure 5E, the repeatability of the samples in each group was favorable. That is, our proteomics data were relatively credible. We concluded that these 62 proteins might play vital roles in the EA-mediated protective effect against MCAO/R-induced cerebral ischemia/reperfusion injury.

### Identification of dysregulated proteins related to brain and nerve development in the MCAO/R v. sham and EA vs. MCAO/R groups

**Figure 6:**
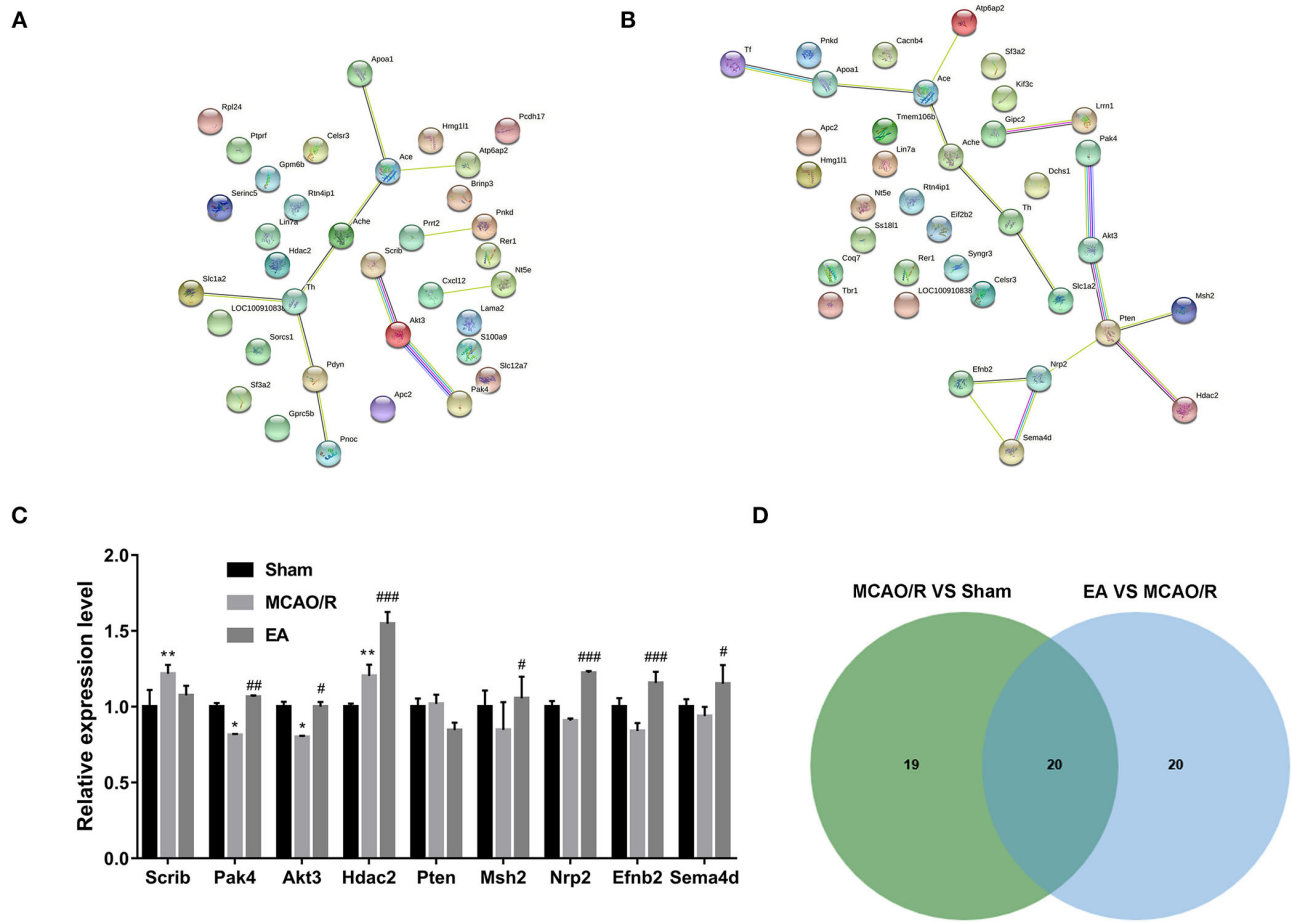
Identification of crucial proteins related to brain and nerve development in response to MCAO/R or EA treatment. **(A)** PPI networks of dysregulated proteins related to brain and nerve development in the MCAO/R vs. sham groups. **(B)** PPI networks of dysregulated proteins related to brain and nerve development in the EA vs. MCAO/R groups. **(C)** Histogram of the proteomics outcomes of Scrib, Pak4, Akt3, Hdac2, Pten, Msh2, Nrp2, Efnb2, and Sema4d in the sham, MCAO/R, and EA groups. **(D)** Jvenn analysis of dysregulated proteins related to brain and nerve development in the MCAO/R vs. sham and EA vs. MCAO/R groups. Each group contained nine hippocampal tissue samples isolated from nine rats, and three samples per group were mixed into a proteomics specimen. Compared with the sham group, *P < 0.05, **P < 0.01; compared with the MCAO/R group, #*P* < 0.05, ##*P* < 0.01, ###*P* < 0.001.

Based on GO biological process enrichment data of differentially expressed proteins in the MCAO/R vs. sham group in Supplementary Table 4, GO biological processes related to brain and nerve development were screened out by searching for the terms containing the keywords brain, neurotransmitter, neuropeptide, neuromuscular, neurogenesis, neuron, nervous, nerve, axon, axonogenesis, synaptic, and synapse. These biological processes are shown in Supplementary Table 5. The differentially expressed proteins in the abovementioned biological processes associated with brain and nerve development are shown in Supplementary Table 6. These dysregulated proteins might be closely linked with MCAO/R-induced brain and nerve damage. Additionally, the potential interactions of proteins as shown in Supplementary Table 6 were predicted using the STRING database. As seen in Figure 6A, serine/threonine protein kinase Akt3 could interact with scribble planar cell polarity protein (Scrib) or serine/threonine protein kinase Pak4 (Figure 6A). Moreover, differentially expressed proteins in the EA vs. MCAO/R model group that might be implicated in brain and nerve development were filtered out based on GO enrichment analysis in Supplementary Table 7 using the abovementioned methods. The screened GO biological process terms and proteins are shown in Supplementary Tables 8, 9, respectively. PPI analysis of these proteins revealed that there were potential regulatory networks among eight proteins [i.e., Pak4, Akt3, phosphatidylinositol 3,4,5-trisphosphate 3-phosphatase and dual-specificity protein phosphatase Pten, DNA mismatch repair protein Msh2, histone deacetylase 2 (Hdac2), neuropilin-2 (Nrp2), ephrin-B2 (Efnb2), and semaphorin 4D (Sema4d)] (Figure 6B). Given the vital roles of the Akt pathway in multiple biological processes and the differential expressions of Akt pathway-related proteins, such as PAK4, AKT3, Pten, Nrp2, and Efnb2, in response to MCAO/R alone or in combination with EA treatment, we hypothesized that AKT pathway-related proteins might be closely linked with MCAO/R-induced brain damage and EA-mediated therapeutic effects against MCAO/R-induced brain damage. Proteomics expression profiles of these Pten/AKT pathway-related proteins in the sham, MCAO/R, and EA groups are shown in Figure 6C. Proteomics outcomes revealed that the Scrib expression level was markedly upregulated (upregulated ratio ≥ 1.2 and *P* ≤ 0.05) in the MCAO/R model group compared with the sham group (Figure 5C). Pak4 and Akt3 expression levels were notably downregulated (downregulated ratio ≤ 0.83 and *P* ≤ 0.05) in the MCAO/R model vs. sham group but markedly upregulated (upregulated ratio ≥ 1.2 and *P* ≤ 0.05) in the EA vs. MCAO/R model group (Figure 6C). Hdac2 was highly expressed (upregulated ratio ≥ 1.2 and *P* ≤ 0.05) in both the MCAO/R model vs. sham group and the EA vs. MCAO/R model group (Figure 6C). The Pten expression level was markedly reduced (downregulated ratio ≤ 0.83 and *P* ≤ 0.05) in the EA vs. MCAO/R model group but was not changed in the MCAO/R model group vs. the sham group (Figure 6C). Msh2, Nrp2, Efnb2, and Sema4d expression levels were markedly increased (upregulated ratio ≥ 1.2 and *P* ≤ 0.05) in hippocampal tissues of rats in the EA group compared with the MCAO/R group (Figure 6C). Jvenn analysis of differentially expressed proteins related to brain and nerve development in Supplementary Tables 7, 9 revealed that there were 20 common proteins in the MCAO/R vs. sham and EA vs. MCAO/R groups (Figure 6D). In addition, 19 or 20 proteins were specifically dysregulated in the MCAO/R vs. sham group or the EA vs. MCAO/R group, respectively (Figure 6D). The expression profiles of these proteins are shown in Supplementary Table 10. Interestingly, converse alteration trends of 15 proteins related to brain and nerve development were observed in the MCAO/R vs. sham and EA vs. MCAO/R groups among 20 common proteins between MCAO/R vs. sham group and EA vs. MCAO/R group (Supplementary Table 10). We supposed that these 15 proteins might play crucial roles in the EA-mediated protective effect against MCAO/R-induced brain injury.

### Western blot validation of Pak4, Akt3, and Efnb2 protein levels in the hippocampal tissues of sham, MCAO/R, and EA rats

**Figure 7:**
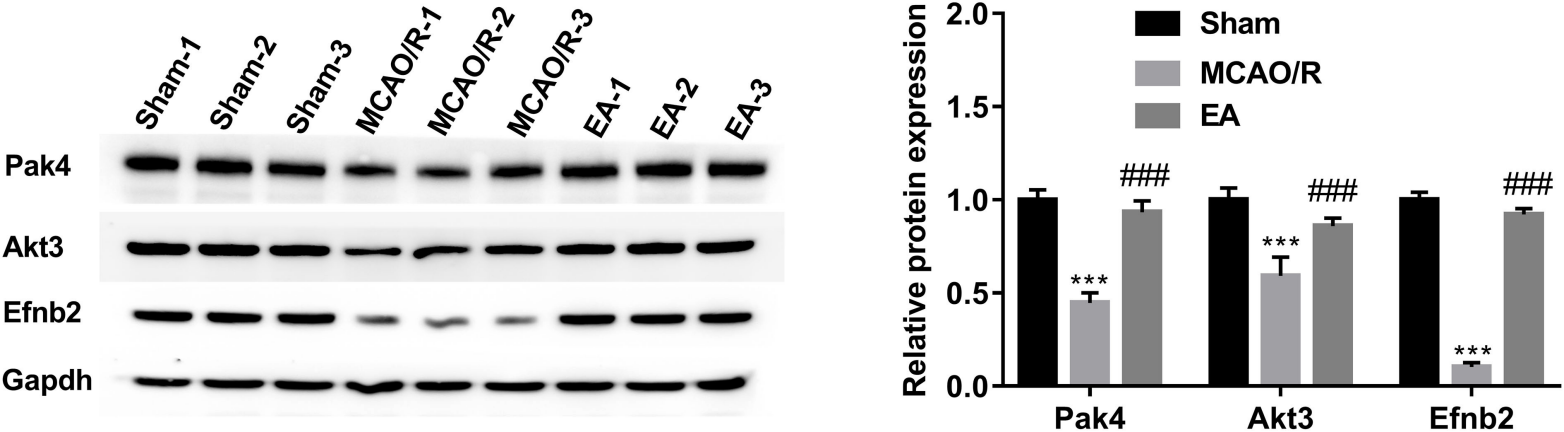
Western blot validation of Pak4, Akt3, and Efnb2 protein levels in hippocampal tissues of sham, MCAO/R, and EA rats. Protein levels of Pak4, Akt3, and Efnb2 were measured by western blot assay in hippocampal tissues of sham (*n* = 3), MCAO/R (*n* = 3), and EA (*n* = 3) rats. Compared with the sham group, ***P < 0.001; compared with the MCAO/R group, ###*P* < 0.001.

The western blot assay demonstrated that the protein levels of Pak4, Akt3, and Efnb2 were markedly reduced in the hippocampal tissues of MCAO/R rats relative to the sham group, while EA treatment abrogated the inhibitory effect of MCAO/R on the expression of Pak4, Akt3, and Efnb2 (Figure 7). The western blot outcomes were consistent with the TMT proteomics results, suggesting that our proteomics data were relatively reliable.

## Discussion

In this study, we demonstrated that EA treatment at the GV20 and GV24 acupoints could markedly alleviate MCAO/R-induced neurological deficits, spatial learning and memory impairments, and cerebral infarction in rats, which was consistent with previous studies (19–21). Moreover, our TMT-based proteomics analysis revealed that 195 proteins were differentially expressed (75 downregulated and 120 upregulated) in the MCAO/R group compared with the sham group. Additionally, 218 differentially expressed proteins (50 downregulated, 168 upregulated) were identified in the EA group compared to the MCAO/R group. Moreover, 62 proteins with converse alteration trends in the MCAO/R vs. sham group and the EA vs. MCAO/R group were screened out. These proteins might be implicated in the EA-mediated protective effect against MCAO/R-induced cerebral injury.

It is well known that ischemic stroke often triggers neuronal and glial injury/dysfunctions, vascular alterations, and inflammation (5, 6). Thus, proteins related to brain and nerve development were further screened out. In our project, 39 differentially expressed proteins in the MCAO/R vs. sham group were implicated in brain and nerve development based on GO enrichment analysis. Additionally, 40 dysregulated proteins in the EA vs. MCAO/R group were identified to be associated with brain and nerve development. PPI analysis of the 39 dysregulated proteins related to brain and nerve development in the MCAO/R vs. sham group suggested that Akt3 could interact with Scrib and Pak4. Additionally, PPI analysis of the 40 dysregulated proteins implicated in brain and nerve development in the EA vs. MCAO/R group showed that there were potential regulatory networks among Pak4, Akt3, Pten, Msh2, Hdac2, Nrp2, Efnb2, and Sema4d. Moreover, proteomics analysis and western blot assays suggested that the protein levels of Pak4, Akt3, and Efnb2 were lower in the hippocampal tissues of MCAO/R rats than in the sham group but were higher in the hippocampal tissues of EA rats than in the MCAO/R group.

Pak4, Akt3, and Efnb2 have been reported to be potential neuroprotective factors. For instance, Won et al. demonstrated that Pak4 could protect dopaminergic neurons and preserve motor function in Parkinson's disease rat models (33). PAK4 loss abrogated the neuroprotective activity induced by the G-protein-coupled receptor 40 agonist GW9508 (34). Aberrant Akt3 expression is closely linked with brain and cortical development and neurogenesis (35–37). Akt3 depletion could trigger the impairment of mouse spatial memory and temporal order discrimination abilities and a reduction in mouse brain size (38, 39). Moreover, previous studies demonstrated that Akt3 was expressed at low levels in the ischemic brain after stroke, and constitutive activation of Akt3 inhibited neuronal death induced by oxygen–glucose deprivation *in vitro* and reduced brain infarction size in a rat stroke model (40, 41). Efnb2, a membrane-tethered ligand of EphB4, can regulate cerebrovascular development *via* the PI3K/Akt pathway (42). Moreover, Ghori et al. pointed out that Efnb2 overexpression facilitated neurovascular repair and alleviated brain injury in the MCAO/R-induced mouse cerebral stroke model (43). Additionally, prior studies showed that Pak4 could positively regulate the PI3K/Akt pathway (44, 45). Furthermore, Akt has been reported to be a potential downstream target of Efnb2 (46, 47). Combined with the PPI, proteomics, and western blot outcomes, we concluded that MCAO/R might induce rat cognitive deficits and cerebral infarction by inhibiting the Pak4/Akt3 and Efnb2/Akt3 pathways and that EA might ameliorate MCAO/R-induced ischemic stroke by activating the Pak4/Akt3 and Efnb2/Akt3 pathways.

Pten is a negative regulator of the Akt signaling pathway, and Pten loss can improve Akt3 activity and expression (48, 49). Pten has also been reported to be involved in the regulation of multiple aspects of brain and nervous system development, such as neuronal polarity, axon/dendrite outgrowth, and myelination (50–52). Moreover, multiple studies suggested that Pten loss or inhibition could notably alleviate OGD/R-induced neuronal injury and MCAO/R-induced ischemic brain injury (53–55). Xing et al. found that EA stimulation at the Quchi (LI11) and Zusanli (ST36) acupoints notably ameliorated neurological deficits and reduced the volume of cerebral infarction in MCAO rats while downregulating the expression level of Pten and upregulating the expression level of Akt, thereby it is speculated that EA exerted its protective effect through the Pten/Akt pathway (56). In addition, Chen et al. observed that EA at the Sishencong (EX-HN1) and Fengchi (GB20) acupoints can downregulate Pten mRNA level, upregulate Akt mRNA level, and improve neuronal apoptosis in the hippocampal tissues of vascular dementia model rats (57). In this study, we found that the Pten expression level was notably reduced in the hippocampal tissues of MCAO/R rats after EA stimulation. Based on these data, we hypothesized that EA treatment at the GV20 and GV24 acupoints could ameliorate cerebral ischemia/reperfusion injury partially by reducing Pten expression and regulating the Pten/Akt3 pathway.

Neuron-specific VEGFR2 knockout inhibited the formation of mature dendritic spines and impaired synaptic plasticity in mice (58). Efnb2 could control VEGFR2 internalization, while VEGFR2 internalization was required for its functions (58). Nrp2, a VEGF receptor (VEGFR), is a vital player in corticostriatal development, goal-directed learning, spine maintenance, hippocampal synaptic plasticity, and GABAergic neuron migration (59–61). Moreover, Goel et al. demonstrated that Pten could notably reduce Nrp2 expression in prostate cancer cells (62). Our proteomics analysis revealed that EA stimulation at the GV20 and GV24 acupoints led to a notable increase in Nrp2 expression levels in the hippocampal tissues of MCAO/R rats. Combined with the PPI prediction data and previous studies, we concluded that Efnb2 might exert its protective effect against MCAO/R-induced brain injury by controlling Nrp2 internalization, and the Efnb2/Nrp2 and Pten/Nrp2 pathways might be implicated in the EA-mediated protective effect against MCAO/R-induced brain injury.

Hdac2 is a crucial factor in many pathophysiologic processes, such as embryonic development, immunity, and proliferation (63). Recently, some studies showed that Hdac2 can impair mouse memory and learning abilities and trigger a reduction in synapse number, synaptic plasticity, and dendritic spine density in the mouse hippocampus (64, 65). Hdac2 expression was markedly increased in the peri-infarct cortex after cerebral stroke (66, 67), and Hdac2 knockdown contributed to mouse motor function recovery after stroke by increasing the expression of neurotrophins and neuroplasticity-related proteins (67). Additionally, Yan et al. demonstrated that Hdac2 could bind to the promoter region of the Pten gene and inhibit Pten expression (68, 69). Our proteomics data revealed that the Hdac2 expression levels were dysregulated in rat hippocampal tissues in response to MCAO/R surgery or EA stimulation, suggesting the potential roles of Hdac2 in MCAO/R-induced cerebral damage and EA treatment of cerebral ischemia/reperfusion injury.

Our proteomics analysis showed that Msh2 and Sema4d were highly expressed in the hippocampal tissues of MCAO/R rats after EA treatment. Msh2, a DNA mismatch repair factor, was notably increased in hippocampal neurons after kainite injection (70). Msh2 depletion caused corpus callosum dysmyelination and locomotive and sensory dysfunctions in mice (71). Previous studies also showed that Sema4d loss could facilitate oligodendrocyte recovery, reduce blood–brain barrier permeability, inhibit the proinflammatory response, curb ischemia-induced cortex cell death, and ameliorate postischemic behavioral abnormalities in the MCAO/R-induced cerebral stroke model (72–74). These data suggested that Msh2 and Sema4d were related to the development of cerebral ischemia/reperfusion injury.

Taken together, our experiments demonstrated that EA stimulation at GV20 and GV24 acupoints notably ameliorated MCAO/R-induced cognitive deficits and cerebral infarction in rats. Moreover, our proteomics outcomes showed that MCAO/R treatment could induce the differential expression of 195 proteins in rat hippocampal tissues. Additionally, 218 proteins were differentially expressed in rat hippocampal tissues of MCAO/R rats after EA treatment at the GV20 and GV24 acupoints. Moreover, 62 proteins with converse alteration trends in the MCAO/R vs. sham group and the EA vs. MCAO/R group were filtered out. These proteins might be involved in mediating the protective effect of EA at the GV20 and GV24 acupoints against MCAO/R-induced cerebral injury. Additionally, 39 dysregulated proteins in the MCAO/R vs. sham group and 40 dysregulated proteins in the EA vs. MCAO/R group were implicated in brain and nerve development. Moreover, our PPI analysis and western blot assay revealed that Pten/Akt pathway-related proteins (e.g., Pak4, Akt3, Pten, Nrp2, and Efnb2) might function as crucial players in the EA-mediated protective effect against MCAO/R-induced brain and nerve injury. Additionally, this study can provide a comprehensive understanding of the molecular basis of EA at the GV20 and GV24 acupoints in the treatment of cerebral ischemia/reperfusion damage.

## Data availability statement

The datasets presented in this study can be found in online repositories. The names of the repository/repositories and accession number(s) can be found in the article/Supplementary material.

## Ethics statement

The animal study was reviewed and approved by the Animal Ethics Committee of the First Affiliated Hospital of Henan University of Chinese Medicine (Approval No. YFYDW2019037).

## Author contributions

XF, JG, and KS designed this study. KS, WH, ZL, MW, JL, YH, and ZZ carried out the experimental research. ZL and MW collected the data. KS and JG performed all analyses. KS and WH wrote the original draft of the manuscript. KS, WH, ZL, MW, JL, YH, ZZ, JG, and XF contributed to the writing of the manuscript. All authors contributed to the article and approved the submitted version.

## Funding

This work was funded by grants from the National Natural Science Foundation of China (Nos. U2004131, 82174473, and 82104973) and the Henan Provincial Science and Technology Department (202102310167).

## Conflict of interest

The authors declare that the research was conducted in the absence of any commercial or financial relationships that could be construed as a potential conflict of interest.

## Publisher's note

All claims expressed in this article are solely those of the authors and do not necessarily represent those of their affiliated organizations, or those of the publisher, the editors and the reviewers. Any product that may be evaluated in this article, or claim that may be made by its manufacturer, is not guaranteed or endorsed by the publisher.
