# Supplementary material for: Electroacupuncture of Baihui and Shenting ameliorates cognitive deficits via Pten/Akt pathway in a rat cerebral ischemia injury model
Source: Front Neurol. 2022 Aug 19;13:855362. doi: 10.3389/fneur.2022.855362 (PMC9437581; doi:10.3389/fneur.2022.855362)
Supplement: Supplementary file 1 [file Data_Sheet_1.docx]

**Supplementary file 1**

**TMT analysis**

**Sample preparation**

Samples were incubated with protein lysis buffer (7M Urea/2M Thiourea/4% SDS/40 mM Tris-HCl, pH 8.5/1mM PMSF/2mM EDTA) for 5 min on the ice. After the addition of DTT (final concentration: 10 mM), cells were sonicated for 15 min on the ice and then centrifuged for 20 min at 13,000×g and 4°C. The supernatants were transferred to new centrifuge tubes and then mixed with four volumes of -20 °C prechilled acetone. The mixtures were kept overnight at -20°C. After centrifuged for 15 min at the conditions of 8,000×g and 4°C, protein pellets were collected, dried in the air and re-dissolved in 8 M urea/100 mM triethylammonium bicarbonate (TEAB) (pH 8.0) solution. Next, proteins were reacted with DTT (final concentration: 10 mM) for 30 min at 56°C and iodoacetamide (IAM, final concentration: 55 mM) for 30 min at room temperature in the dark. Next, protein concentration was determined using the Bradford method. After diluted 5-fold with 100 mM TEAB, proteins (100 μg/sample) were digested overnight with trypsin at an enzyme-protein ratio of 1:50 (w/w) at 37°C. The peptide segments after enzymolysis were desalted using C18 columns and then dried using the vacuum freezing method.

**TMT labeling**

Next, the dried peptides were redissolved in 0.5 M TEAB solution, and then labeled using the TMT label reagent (Thermo Scientific, Waltham, MA, USA) following the protocols of the manufacturer. Samples were labelled as follows: C1 (sham group), 126; C2 (sham group), 127N; C3 (sham group), 127C; M1 (MCAO/R model group), 128 N; M2 (MCAO/R model group), 128C; M3 (MCAO/R model group), 129N; T1 (EA treatment group), 129C; T2 (EA treatment group), 130N; T3 (EA treatment group), 130C. Next, mixed peptides were fractionated by increasing Acetonitrile (ACN) concentration under the alkaline condition on Ultimate 3000 HPLC system (Thermo Scientific) coupled with Durashell C18 column (5μm, 100 Å, 4.6 × 250 mm). Distillates were collected each minute and merged into 10 fractions.

**[Liquid](https://cn.bing.com/dict/clientsearch?mkt=zh-CN&setLang=zh&form=BDVEHC&ClientVer=BDDTV3.5.1.4320&q=%E6%B6%B2%E7%9B%B8%E8%89%B2%E8%B0%B1-%E8%B4%A8%E8%B0%B1%E8%81%94%E7%94%A8%E5%88%86%E6%9E%90)** **[chromatography](https://cn.bing.com/dict/clientsearch?mkt=zh-CN&setLang=zh&form=BDVEHC&ClientVer=BDDTV3.5.1.4320&q=%E6%B6%B2%E7%9B%B8%E8%89%B2%E8%B0%B1-%E8%B4%A8%E8%B0%B1%E8%81%94%E7%94%A8%E5%88%86%E6%9E%90)****[-](https://cn.bing.com/dict/clientsearch?mkt=zh-CN&setLang=zh&form=BDVEHC&ClientVer=BDDTV3.5.1.4320&q=%E6%B6%B2%E7%9B%B8%E8%89%B2%E8%B0%B1-%E8%B4%A8%E8%B0%B1%E8%81%94%E7%94%A8%E5%88%86%E6%9E%90)****[mass](https://cn.bing.com/dict/clientsearch?mkt=zh-CN&setLang=zh&form=BDVEHC&ClientVer=BDDTV3.5.1.4320&q=%E6%B6%B2%E7%9B%B8%E8%89%B2%E8%B0%B1-%E8%B4%A8%E8%B0%B1%E8%81%94%E7%94%A8%E5%88%86%E6%9E%90)** **[spectrometry](https://cn.bing.com/dict/clientsearch?mkt=zh-CN&setLang=zh&form=BDVEHC&ClientVer=BDDTV3.5.1.4320&q=%E6%B6%B2%E7%9B%B8%E8%89%B2%E8%B0%B1-%E8%B4%A8%E8%B0%B1%E8%81%94%E7%94%A8%E5%88%86%E6%9E%90) (MS) analysis**

After lyophilization, samples were isolated and analyzed using Q Exactive HF-X Mass Spectrometer (Thermo Scientific) and UltiMate 3000 RSLCnano Liquid Chromatography system (Thermo Scientific). Peptides were captured by C18 capture column (3 μm, 120 Å, 100 μm × 20 mm) and eluted on analytical columns (2 μm, 120 Å, 750 μm × 250 mm). Mobile phase A was composed of 3% DMSO, 0.1% formic acid, and 97% H_2_O, and mobile phase B was composed of 3% DMSO, 0.1% formic acid, and 97% acetonitrile. The flow rate of liquid phase was set as 300 nL/min. In the DDA analytical pattern, each scan cycle contained a MS full scan (R = 60 K, AGC = 3e6, max IT = 20 ms, scan range = 350-1800 m/z) and 20 MS/MS scans (R = 15 K, AGC = 2e5, max IT = 100 ms). HCD collision energy was set as 28. The filter window of quadrupole was set as 1.6 Da. The dynamic exclusion time of ion repeat collection was set as 35s.

**Protein identification**

Proteins were identified using MaxQuant v1.6.0.1. Contaminated proteins, anti-library proteins, and proteins that were identified only by modification sites were removed from our project. Proteins with at least one unique peptide and FDR ≤ 1% were included in the subsequent analysis.

The retrieval parameters of MaxQuant were shown as blow.

| **Item** | **Value** |
| --- | --- |
| **Type** | Reporter ion MS2 |
| **Isobaric labels** | Reporter Quantification (TMT 10 plex) |
| **Enzyme** | Trypsin/P |
| **Instrument** | Thermo Q Exactive™ HF-X |
| **Max. Missed Cleavage Sites** | 2 |
| **Precursor Mass Tolerance** | 10 ppm |
| **Fragment Mass Tolerance** | 0.02 Da |
| **Fixed modifications** | Carbamidomethyl(C) |
| **Variable Modifications** | Oxidation (M), Acetyl (Protein N-term), Gln->pyro-Glu |
| **Instrument type** | Orbitrap |
| **Match between runs**  **Other parameters** | Clicked  Default values |

**Protein quantification**

Proteins were quantified using the Maxquant software. Differences between groups were compared using Student’s t test. *P* values obtained from Student’s t test were corrected through false discovery rate (FDR). The values after FDR correction were termed as Q value.

**Differential expression analysis**

Proteins (up-regulated ratio ≥1.2 or down-regulated ratio ≤ 0.83 and Q value ≤ 0.05) were considered to be significantly differentially expressed.

**Annotation and enrichment analysis**

To determine the biological and functional properties of all identified proteins, the identified protein sequences were mapped with Gene Ontology Terms (<http://geneontology.org/>). For this, homology search was first performed for all the identified sequences with a localized NCBI blastp program against NCBInr animal database. The e-value was set to less than 1e^-5^, and the best hit for each query sequence was taken account for GO term matching. GO enrichment analysis was performed using hypergeometric test.

**References**

1. Conesa A, Götz S: **Blast2GO: A comprehensive suite for functional analysis in plant genomics**. *International journal of plant genomics* 2008, **2008**:619832.
